# Migration of Over-the-scope Clip Resulting in Anal Pain and Obstructed Defecation

**DOI:** 10.7759/cureus.7572

**Published:** 2020-04-07

**Authors:** Sameen Khalid, Jinendra Satiya, Aamer Abbass, Gulshan Parasher, Daniel Castresana

**Affiliations:** 1 Gastroenterology and Hepatology, University of New Mexico, Albuquerque, USA; 2 Internal Medicine, NYC Health + Hospitals/Metropolitan, New York, USA

**Keywords:** polyps, adenomas, endoscopic resection, polypectomy, over-the-scope clips

## Abstract

Iatrogenic perforation is a known and feared complication of diagnostic and therapeutic colonoscopy. Specific locations in the gastrointestinal tract, such as the jejunum, have a higher risk of perforation owing to its difficult anatomical position. Over-the-scope clips have recently been used for the management of these perforations. We present the case of a 40-year-old male patient treated with over-the-scope (Ovesco^®^, Ovesco Endoscopy AG, Tübingen, Germany) clips for an iatrogenic postpolypectomy perforation with subsequent anal pain and inability to evacuate stool occurring as a result of the migration of the clip, followed by a review of the literature.

## Introduction

Over-the-scope clips (OTSCs) have been increasingly used in the management of gastrointestinal bleeding, iatrogenic perforations after polypectomy, and anastomotic fistulas [1,2]. Iatrogenic perforation during a diagnostic or therapeutic colonoscopy is a known complication with an incidence ranging from 0.1% to 0.3% of cases. These complications carry a high morbidity and mortality rate and, in the past, warranted surgical intervention [3,4]. However, with the advances in therapeutic endoscopy, many perforations can be managed effectively with endoclips, OTSCs, and/or endosuturing. These new techniques come with device-specific risks, and Fischer et al. reported a case of OTSC-induced bowel obstruction [5]. Here, we present a case of migration of OTSC placed for postpolypectomy perforation resulting in anal pain and obstructed defecation.

## Case presentation

**Figure 1:**
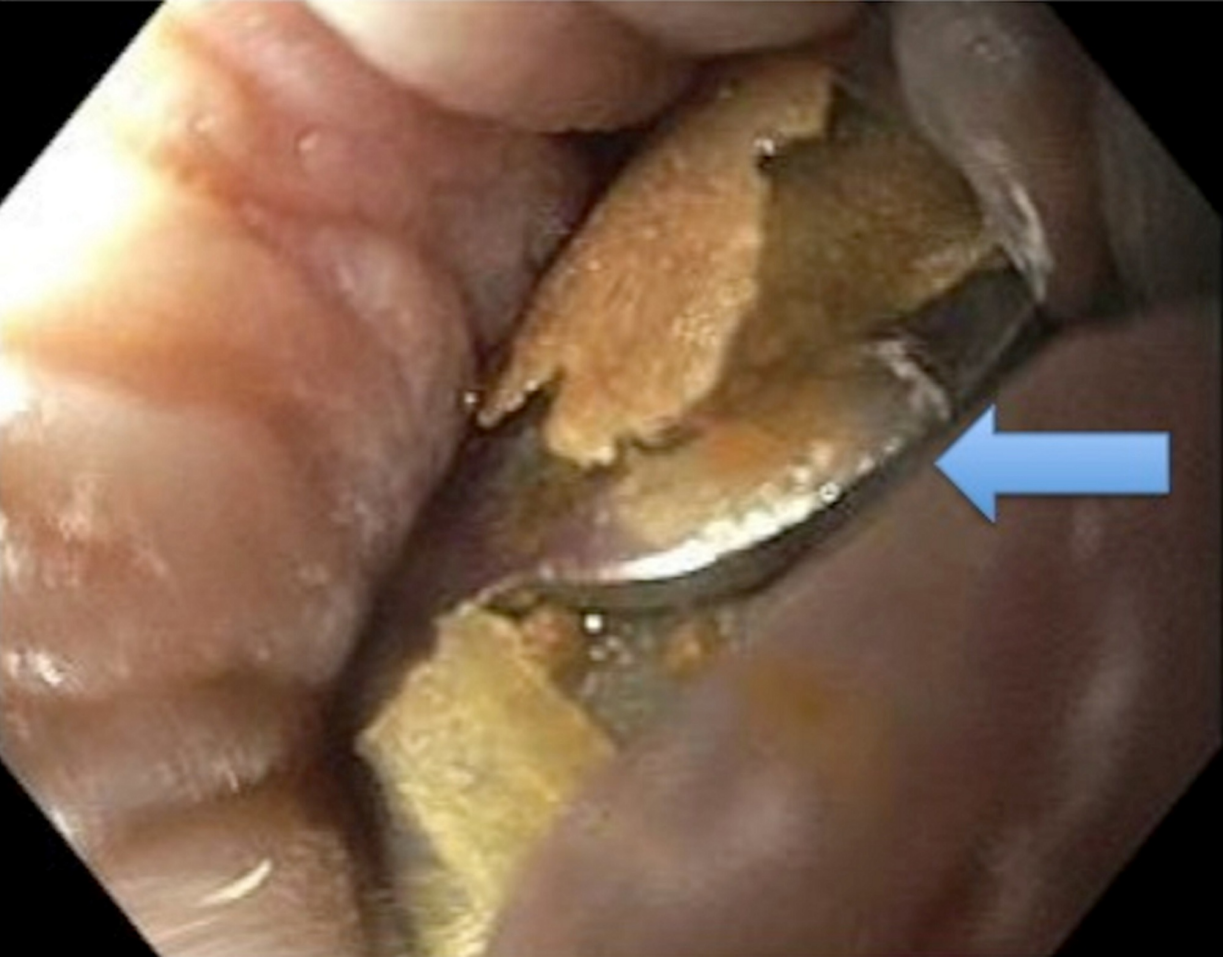
Two over-the-scope clips seen on endoscopy approximately 2 cm from the anal verge embedded in the mucosa

A 40-year-old male patient with a history of familial adenomatous polyposis underwent total proctocolectomy, ileoanal anastomosis, and creation of a J pouch. The patient had a pouchoscopy performed for polyp surveillance, and a 3-cm flat polyp located 30 cm from the anal verge in the pouch was removed with a piecemeal technique using a spiral hot snare and Eleview® (Medtronic, Dublin, Ireland) injection lift technique. Subsequently, a hole-like defect was noted in the postpolypectomy site that developed into an obvious small 2-3 mm perforation. To repair the defect, the tissue edges were approximated, and one 14/6-mm type gastric fistula closure (gc) OTSC (Ovesco®, Ovesco Endoscopy AG, Tübingen, Germany) was placed with successful closure of the defect. To repair the residual diminutive defect, the remaining tissue edges of the mucosal defect were approximated, and another 12/6-mm type gc OTSC (Ovesco®) was successfully placed. With the two OTSCs, no residual defect or bleeding was noted at the postpolypectomy site. Four months later, the patient presented to the emergency department with severe anal pain while attempting to have a bowel movement. He then tried to perform manual disimpaction without success and felt a hard, metallic object in the anal canal. He denied any fevers, chills, nausea, vomiting, or abdominal pain. On physical examination, he was hemodynamically stable. Abdominal examination revealed no abdominal tenderness, and bowel sounds were normal. Anal tenderness was found on digital examination. Routine laboratory testing results, including complete blood counts, were unremarkable. A computed tomography (CT) of the abdomen and pelvis did show an object of metallic density at the anal verge with non-obstructive bowel gas pattern. Endoscopy under conscious sedation revealed two OTSCs approximately 2 cm from the anal verge embedded in the mucosa (Figure 1).

The endoscope could not be advanced past the clips, and the clips could not be removed due to the patient’s intolerance of the procedure because of significant pain. The patient underwent examination under anesthesia thereafter with removal of two OTSCs from the ileoanal pouch. Endoscopy was subsequently performed with an evaluation of the mucosa that appeared to be normal. The patient did well postprocedure and was discharged home the next day. 

## Discussion

Ovesco® was first approved in Europe in 2009 and secured approval in the United States in 2010 [6]. The Ovesco® clip is a novel endoscopic method to achieve mechanical compression of gastrointestinal tissue. Current indications include closure of fistulas and iatrogenic perforations, hemostasis, non-variceal gastrointestinal bleeding not responding to conventional endoscopic treatment, and closure of the submucosal tunnel made after peroral endoscopic myotomy, endoscopic submucosal dissection, or after natural orifice transluminal endoscopic surgery [3]. Another promising application is the anchoring of fully covered metal esophageal stents to prevent migration [7]. It is a biocompatible device made from nitinol with self-memory shape. The technique is similar to variceal band ligation and can easily be learned by a general gastroenterologist. 

Experience with Ovesco® clips is restricted to case reports and retrospective studies. Angsuwatcharakon et al. retrospectively evaluated the use of Ovesco® clips to treat iatrogenic perforations in a series of six patients from three tertiary care hospitals in Thailand [8]. They reported a technical success rate of 100%, with a clinical success rate of 83.3%. Five perforations were in the duodenal wall and one in the rectosigmoid junction. The average size perforation size was 13 mm. They reported no mortality and proposed Ovesco® as the preferred method for perforation closure, especially in lesions smaller than 30 mm. Parodi et al. delineated the successful use of Ovesco® clips for the management of leaks secondary to perforations, anastomotic dehiscence, and fistulas in a series of 10 patients [9]. Samarasena et al. first described the use of Ovesco® clips to treat a jejunal perforation, an area with a thinner wall, and a high rate of perforation [10]. Purchiaroni et al. recommended the use of the OTSC system, premounted onto a double-balloon enteroscope, as a safe alternative to conventional metal clips for fast closure of jejunal perforations [11].

Over-the-scope-clips have been used in a variety of settings with good results. Manta et al. achieved hemostasis in 29 of 30 cases (97%) of upper and lower gastrointestinal bleeding unresponsive to conventional endoscopic treatment [12]. Disibeyaz et al. reported satisfactory results in 21 of 22 cases of perforations [13]. Soria et al. successfully used the Ovesco® clip to control bleeding from a Dieulafoy lesion [14]. Caputo et al. successfully used the remOVE system (Ovesco Endoscopy AG, Tübingen, Germany) for OTSC clip removal in 72 of 74 patients (97.3 %) [15].

OTSCs can be used with blunt teeth (type a) in non-fibrotic tissues for a primarily compression effect. For chronic ulcers and fistulas, application aids such as a grasper or OTSC with sharp teeth (traumatic version [type t] or type gc) are recommended [7]. Complications after placement of an Ovesco® clip are rare. Nishiyama et al. reported no complications at seven days post-treatment with an Ovesco® clip [16]. Alastal et al. reported a case of acute cholangitis after a duodenal OTSC placement for a chronic duodenocutaneous fistula [17]. Voermans et al. reported one case of esophageal perforation, which they attributed to the introduction of the endoscope with the OTSC in their prospective multicenter study [18]. Surace et al. reported one complication of a blocked anchor. The anchor was blocked within the clip, not allowing for immediate withdrawal at the time of placement of the OTSC. The clip was subsequently removed one week later [19]. Fischer et al. reported the first case of mechanical clip-induced bowel obstruction [5]. This occurred with a 14 mm/6 mm type t OTSC that was placed for an iatrogenic perforation in a 75-year-old male patient who underwent diagnostic colonoscopy for evaluation of chronic anemia and was found to have sigmoid diverticulosis with signs of postinflammatory bowel-wall sclerosis. They noted the colonic lumen to be considerably narrowed after the placement of the OTSC. Given the clinical signs of obstruction and a CT scan with contrast enema depicting total blockage of flow at the level of the OTSC, the patient underwent a sigmoid resection two days later. They found the clip causing a visible tissue bulge, and a diagnosis of mechanical bowel obstruction was made. In our case, an Ovesco® clip was used to successfully treat a 2-3 mm postpolypectomy perforation in a J pouch. The patient presented four months later with clinical signs of anal pain and obstructed defecation. An endoscopy revealed two OTSCs approximately 2 cm from the anal verge embedded in the mucosa. The patient was treated with surgical intervention due to inability to pass the scope beyond the clips. The patient did well with no complications and was discharged home the next day. 

This work has been presented as an abstract at American College of Gastroenterology Annual Meeting, San Antonio, TX (Khalid S, Satiya J, Abbass A, Parasher G, Castresana D. Migration of Over-the-Scope Clip (OTSC) Resulting in Intestinal Obstruction; October 28, 2019).

## Conclusions

The Ovesco® clip is a novel endoscopic treatment option for closure of iatrogenic perforations. It has been proven to have a high success rate. All gastroenterologists should be aware of clinical scenarios in which the Ovesco® clip can be used, especially non-variceal gastrointestinal bleeding non-responsive to conventional endoscopic treatment. Complications after Ovesco® clip placement are rare but important and may include mechanical bowel obstruction, mechanical anal obstruction, acute cholangitis, and perforation during its introduction into the bowel lumen. Clinicians should be cognizant and vigilant of these complications while using the Ovesco® clip. Comparative studies involving a large set of patients with long-term follow-up are required.
